# Cultural and creative sectors at a crossroad: from a mainstream process towards an active engagement

**DOI:** 10.1186/s43238-021-00032-y

**Published:** 2021-09-02

**Authors:** Christian Ost, Ruba Saleh

**Affiliations:** 1 Raymond Lemaire International Conservation Centre, KU Leuven University, Kasteelpark Arenberg 1, B –3001 Heverlee, Belgium; 2 ICHEC Brussels Management School, Brussels, 4 Boulevard Brand Whitlock, 1150 Brussels, Belgium

**Keywords:** Cultural and creative sectors (CCS), Cultural heritage, Cultural entrepreneurship, Long wave theory, Sustainable and circular business model, COVID-19, Cultural resilience

## Abstract

The COVID-19 pandemic has led to a current global health crisis with dreadful repercussions all over the world. A global economic recession is anticipated, with strong impacts in all economic and social sectors, including the cultural sector. Although all sub sectors will be impacted (heritage sites, theatres, museums, operas, art galleries), the cultural built heritage is particularly at stake, as it relies on multiple stakeholders through a wide range of heritage-related activities (tourism, recreation, housing, real estate, construction, craftsmanship, etc.). Sites management and heritage conservation have not only been vulnerable to strong economic and social disruptions, like most of other cultural fields, but have been greatly challenged because heritage values and the paradigm of conservation (50 years after adoption of the UNESCO convention) are being themselves revisited in the perspective of the Sustainable Development Goals. The paper aims also to consider cultural heritage as part of the Cultural and Creative Sectors (CCS) and how creativity and innovation contribute to post-COVID recoveries through Schumpeter-related creative destruction process. The current crisis might be perceived in a perspective of long wave theory of innovations and economic growth. The economic history is filled with many examples of such transition period when inventions, innovations, and growth reactivate the economic development in an upward long-term trend. In such framework, crisis can trigger innovation and creativity and can be understood as opportunity to increase the CCS resilience and sustainability, as well as harness the universality and the power of creativity. Finally, the paper aims to describe implications of such situation by providing to the CCS ways to learn and experience cultural entrepreneurship, resilient strategies, new sustainable and circular business models applied to the cultural heritage sector and its conservation.

## Introduction

The current global health crises have dreadful repercussions all over the world, not just anticipating a new global economic recession, but a severe transition in the long-run with all economic and social sectors being challenged and impacted by the COVID-19 pandemic. As it can be expected from a sanitary crisis, it impacts more the urban context with its variety of cultural activities and immovable built heritage, with heritage sites, theatres, museums, operas, art galleries and other places of public congregation being forced to close. Festivals, fairs, performances, biennales and concerts have been cancelled. Public and private cultural institutions and foundations, numerous freelancers in heritage conservation, performing arts, film and music, and related businesses have seen months, if not years, of intense creative effort and preparation evaporate at short notice. In terms of industries, the Cultural and Creative Sectors^1^ (CCS) have been affected more severely than any other, being positioned between a very elastic demand-sided consumption, and still fragile innovative supply-sided industries.

The challenge is global, the solutions are diverse and adjusted to local and sectoral considerations. As far as the CCS is concerned, some countries and public agencies provided support to institutions and individuals, but not enough (IDEA Consult et al. 2021). Durable solutions go beyond quantifiable or one-shot financial support. In addition, entrepreneurs and small- and medium-sized enterprises, which often lack the resources to respond to an emergency of this magnitude, are especially vulnerable. Freelancers, part-timers and gig workers, who make up a large segment of the sector’s labour force, are left with limited to no access to conventional social protection mechanisms (Gill and Pratt 2008; Pratt 2017; Liemt and Gijsbert 2014). Part of the cultural and creative labour force relies on a shadow economy and does not appear in the official accounts and databases. Instability and economic uncertainty in the CCS is big, and the global health crisis unpacks and intensifies the CCS foregoing precariousness (Comunian and England 2020).

In fact, the crisis heavily impacts the creative value chain (creation, production, dissemination, distribution and engagement) and significantly hinders the professional, social and already volatile economic status of artists and cultural and creative professionals. It is clear that, as with the rest of the economy, emergency plans are needed, the possibility of accessing extraordinary resources in order to avoid a destruction of the cultural capital on a large scale and give the necessary time to cultural institutions and operators for experimenting with new forms of sustainability, to face the most difficult challenge ever: overcome in one leap the whirlpool that suddenly opened and recover structural weaknesses with more solid foundations.

The survey conducted by ICOMOS in 2020 on the impact of COVID-19 on heritage found that tangible, intangible and natural heritage were heavily impacted by the pandemic. However, it also highlighted that ‘heritage sites and objects emerged as non-renewable resource for human social, economic, cultural, and moral well-being … heritage has an impact on human rights, equality, accessibility, humanity, identity and diversity’ (ICOMOS 2020, 63). The cultural built heritage has a privileged position in such a creative value chain. Being the locus of pluri-disciplinary activities, it encompasses intrinsic and extrinsic values, use and non-use values, direct and indirect impacts, individual and collective needs, private and public economic good considerations *‘*heritage value typologies … describe the same pie, but slice it in subtly different ways*’.* ***(***Mason 2002, 10).

## A Schumpeterian perspective to CCS and cultural heritage

Despite the fact that creativity relied initially to Arts and Culture, its meaning has extended well beyond the artistic and cultural field and is today recognised as a fundamental component of economic development. But this is not just about creativity, it is about creative ‘industries’. The emergence of the CCS demonstrated that the scope of cultural creativity has extended to a wider range of production and consumption behaviours and processes. Industries (or economic sectors) are supply-sided units which provide products and services on the different markets, and it is acknowledged that CCS are among the most recent and booming industries with a lot of potential in development (KEA and PPMI 2019). ‘In 2017, there were more than 1.1 million cultural enterprises in the EU-27, representing approximately 5 % of all enterprises within the non-financial business economy. Together they generated a total value added of more than EUR 145 billion, equivalent to 2.3 % of the total non-financial business economy’ (IDEA Consult et al. 2021, 15).

Economic development is not and has never been a linear process. It has been the result of growing trends in prices and production, peaks, slumps and financial crisis, long transitions from old to new technologies, and mostly, recurrently cyclical changes (Dupriez and Ost 1996). Apart from wars and other exogenous elements, the first systematic explanations for such long-term cyclical changes (called hereunder long waves) were sought in monetary facts. Thanks to institutional changes in the monetary and financial systems over time, long waves could develop by adjusting money supply to financing needs in strong, and in weak phases of growth, the occurrence of financial crisis being the most visible signs of such adjustments (L. H. Dupriez 1951). Other explanations have been suggested, like the acceleration and deceleration in the rate of capital accumulation which is the reason for disruptive moves between demand and supply (Mandel 1980). Such explanations contribute to a systemic vision of the economy and society, where any human activity will be impacted by a cluster of challenges, related to either the creation and production of goods and services, or their dissemination and consumption. As far as the built cultural heritage is concerned, there is historic evidence for cyclical changes both on supply-side (waves of construction and destruction or reconstruction that includes new forms of heritage replacing ancient ones) and on demand-side (waves of functions and uses related to changing needs and consumers’ behaviour). This appears especially in urban context where urban transformations are not linear, but a narrative of historic turmoil, prosperity, crisis peace, wars and revolutions. Replacement of buildings, changes in architectural styles, and transformation of urban needs contribute to such historic and fractured urban metabolism. Each of the explanations are correlated to another key phenomenon which has been highlighted since the very start of economic research on long waves: the central role of technological innovations.

Not only do innovations appear massively in the low growth phase (their large-scale use only taking place at the start of the next strong growth phase), but they are characterised by an endogenous component specific to long wave theory. This is due to the distinction, already made by Kondratieff, between invention and innovation. ‘Scientific-technical inventions in themselves, however, are insufficient to bring about a real change in the technique of production. They can remain ineffective so long as economic conditions favourable to their applications are absent’ (Kondratieff and Stolper 1935). Later, Joseph A. Schumpeter revisited the central role of innovation in any process of growth in the capitalist world, and more particularly in the various types of fluctuations that compose it. In the case of long wave theory, the explanation would lie in the discontinuities that characterize the appearance, impact and diffusion of innovations throughout the economy over time (Schumpeter 1939).

As a subsequent and similar concept of artistic creativity, economic innovation is defined as the risk-taking behaviour from capitalists and entrepreneurs when introducing new technological inventions on the market. These timely decisions turned challenges into opportunities and have benefited from the favourable economic conditions at the upturn of the economic wave (idle resources and labour force, excess saving and money supply, absence of inflation). Schumpeter’s approach emphasises not only the importance of innovations, but also those of entrepreneurs who must clearly be distinguished from managers. When cultural activities are at stake, the same distinction should be made between cultural entrepreneurs (artistic creativity) and cultural managers (operational tasks).

Still, even if the entrepreneur is sublimated in Schumpeter’s theory, there is no explanation on why innovations appear at certain times and usually in large numbers (clusters) (Thomas 1984). To answer this question, a new interpretation of the emergence of innovations has been suggested, giving a key-role to basic innovations that create new products, new sectors, new markets. Basic innovations do not appear on a permanent basis, or in a random mode; they appear in clusters during the weak phase of the long wave. Gerhard Mensch has suggested to consider the weak phase of the long wave as a technological dead end (*das technologische Patt*), characterised by the absence of creativity, of cultural initiatives, of risk-taking decisions (Mensch 1979). On the opposite, the weak phase challenges entrepreneurs to provide a reorientation by means of a new wave of basic innovations (*Innovation berwinden die Depression*). This state-of-mind and entrepreneurial spirit regenerates the economy by creating new ideas, new products and services, as the foundations for the new long wave. The continuing process is made up of the replacement of basic innovations by improvement innovations.

As far as the long wave is characterised, product innovations come first by adapting scientific inventions to consumption needs (example: new techniques of conservation, new material for artistic creations). Then, process innovations come, as new products and services will expand to all the consumers’ brackets, to all geographical areas, and to all subsections of the cultural and creative industries. Finally, organisational innovations come, with new business and governance models to adjust to the new organization of the economy. The probability of a new wave of innovations coming with sustainability-centred challenges will further require organisational changes in the enterprises impacted by a decrease in productivity and loss of competitiveness (Silva and Serio 2016).

This later stage may take some time to be implemented (sometimes a whole generation), since its success is highly correlated to changes in education, knowledge and skills, also to the social turmoil and resilience vis-à-vis the destruction of obsolete jobs and industries that accompanies the creation of new jobs and industries. In arts and culture, like in economics, creative destruction is the result of time that goes by, with modes and styles, winners and losers, individuals from the past and newcomers. It will thus, require a change of mind in the behaviour and skills of the cultural actors.

**Fig. 1:**
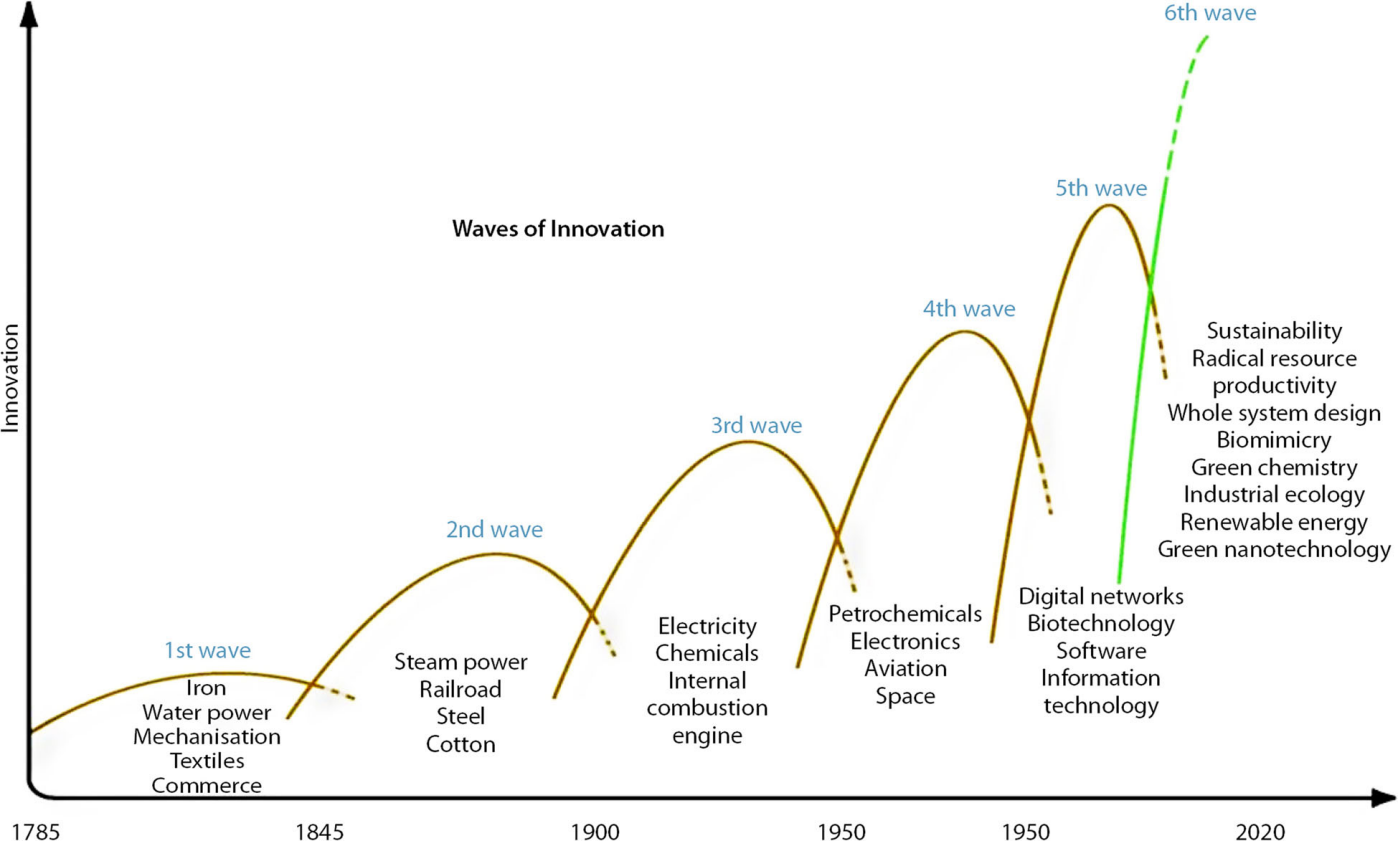
Waves of innovations along the economic development. (Source: Hargroves and Smith 2005, 17)

Figure 1 is a tentative display of waves of innovation as described initially by Nicholas Kondratieff, and subsequentially by Joseph Schumpeter. It depicts six long innovation waves that have contributed to long term development (the last one is a prediction). The first waves are related to the industrial revolution. The two last waves highlight the environmental concern and the subsequent sustainability issue for economic development that are mainstream these days. It should be reminded that long-wave theory is based on rupture, crisis, and recoveries. When easily explainable at the top of the wave (downwellings are the result of upwellings), it still is unclear how waves start to recover, reconstruct, rebirth … Kondratieff suggested that major unexpected world events (wars, revolutions, catastrophes, etc. …) could explain the upwards movement. It is not impossible to see the COVID-19 worldwide crisis such as one of these ‘catalysts’ that are considered in long-wave theory (Narkus 2012).

**Fig. 2:**
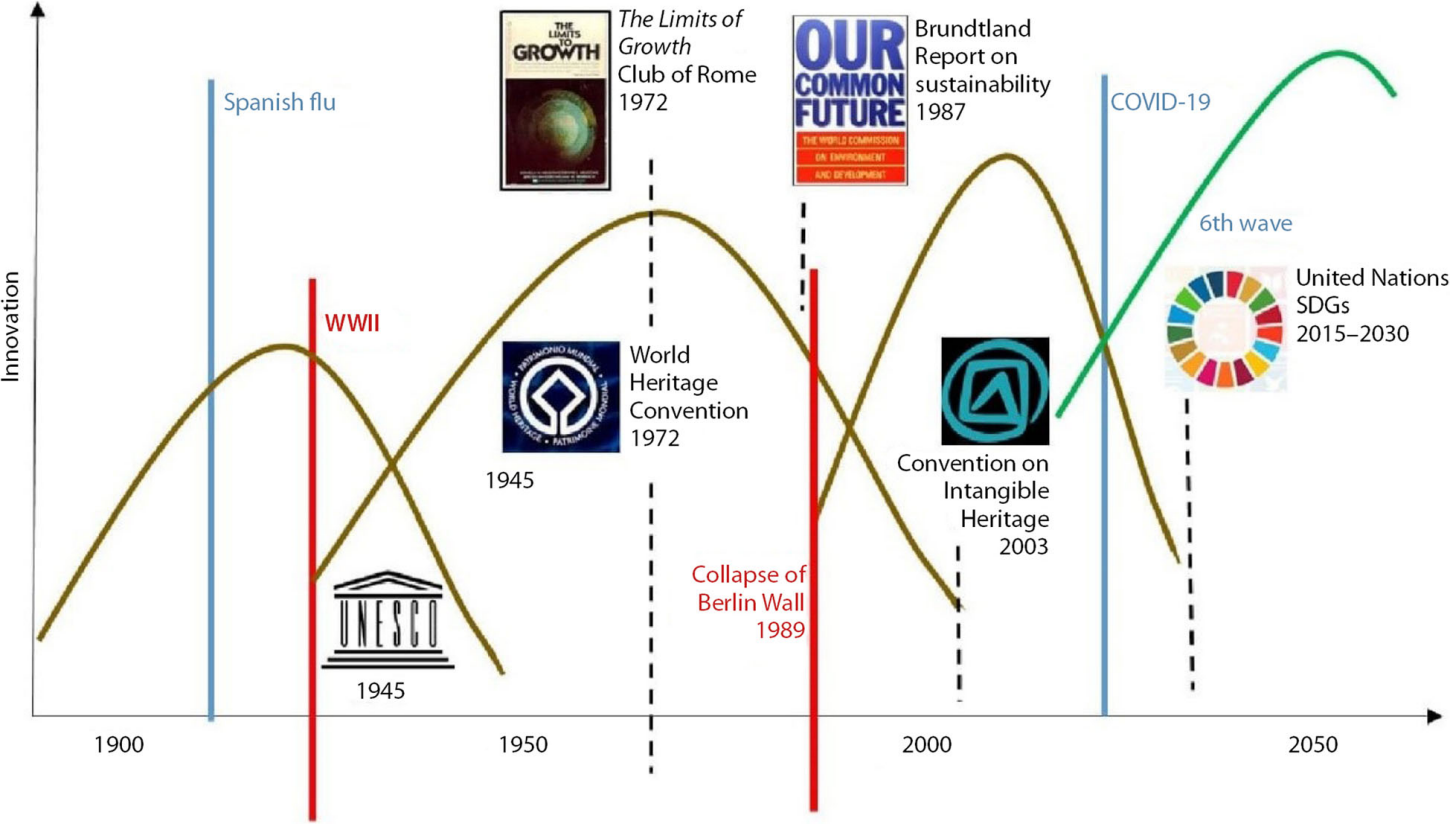
Waves of innovation with the cultural built heritage (Source: the authors)

A closer look to economic waves during the last century is highlighted in Fig. 2. It aims to explain the role of culture, and in particular of cultural tangible and intangible heritage. Indeed, modern conservation is timely related to the last waves in Fig. 1. The successful story of heritage conservation during the last 50 years is an indication of the strong correlation between protection/conservation of cultural built heritage, and structural factors emerging after WW2. The 4th wave has provided in Europe not just the awareness towards heritage given the substantial WW2 tangible heritage destructions, but also the conditions for rebuilding, protecting and developing the heritage as recognized cultural built assets. During the Golden Sixties, both supply-side incentives and new demand-driven consumer’s behaviours for travelling and visiting the heritage have contributed to the emergence of new industries. The next wave has experienced new digital technologies and growing concern for the environmental issues. Globalisation that has followed the collapse of the Berlin wall in 1989 has provided to cultural heritage the best and the worse, with a surge of economic opportunities from cultural tourism, and the emergence of threats from aggressive economic development, in particular in urban settings (Ost 2021). The sector has to manage its own success and its fragility in an economic system that privileged privatisation, financialisation, and market dominance. The UNESCO urban sustainable agenda fits perfectly into this framework, in responding to the United Nations Sustainable Development Goals aimed to sustainability and inclusiveness (UNESCO 2016). In such a framework of threatening challenges and new available technologies, the cultural heritage field must be creative enough to innovate in terms of products (like extending the cultural heritage to more intangible components), in terms of processes (like 3-D virtual visits for cultural tourism), and in terms of organizations (like new governance models, public private partnerships). The list of creative innovations that can be applied to the cultural sector at large (and to all subcategories in the CCS) has no limit, and it would be useful (although it is not the purpose of this paper to do that) to sort and categorize the cultural creativity in terms of long wave movements and future growth.

In particular, the two last waves (the very last one being prospective) would provide innovations which benefit over time to all economic sectors. Some of these innovations may substantially contribute to growing cultural and creative industries too. The Schumpeterian perspective remains strong today, where entrepreneurship and innovations are key-elements in the post COVID-19 recovery of the global economy. Current innovation theories enlarge the concept of entrepreneurship to a more systemic role, and stress on the dynamics between different actors and stakeholders. ‘a much more complex phenomenon whereby changes in one component in a system trigger changes in other components’ (Vandesande 2017). As per the CCS, innovations will be conducted by a group of entrepreneurs, and ultimately by a community on local or global level. Such organisational changes are duly integrated with innovative approaches like the Historic Urban Landscape, a systemic urban conservation approach which stresses on strong community and capacity-building techniques, and generates new and diverse forms of heritage preservation in terms of business and governance models (Bandarin and van Oers 2012; Bandarin and Van Oers 2014; Pereira Roders and Bandarin 2019; UNESCO 2011).

## Education and learning as long-term recovery strategies

The post COVID-19 economic transition will not be only a cyclical crisis but a structural crisis, with very high level of uncertainty and long-term challenges that may impact the economy and the society as a whole. Long wave theories specifically address the issue of parallel trends in education and economic growth on the premises that innovations are based on generation*-*learning models (Devezas and Linstone 2005). The creative destruction process that is part of the transition of the economy towards a new wave of strong growth based on basic and improvement innovations, explains the shift between old and new technologies, old and new industries, old and new resources allocation that include jobs.

Economic and social conditions to achieve this delicate transition are manifold, like the market flexibility, the existence of public support policies, institutional rigidities or market asymmetries (cyclical upturns and downturns do not react in similar way), social resilience to changes, cultural behaviours in terms of risk-taking and individual responsibility, learning and education systems, the latter two being at stake in a training program of cultural entrepreneurship.

The cultural sector, and in particular the cultural heritage, is no exception to the conditions for successful recovery. As far as artistic and cultural activities are concerned, individual skills and know-how are key-resources, and the generally labour-intensive process of such activities is about to explain weak productivity gains. Baumol’s law states that cultural sectors experienced no or low increase of labour productivity thus, undergo unwelcome cost increase in response to rising salaries (W. Baumol and Baumol 1986; W. J. Baumol 1967; Hobgood 1967). While the recovery benefit to capital-intensive industries at an early stage because the shift of resources is easy to be implemented, the CCS lacks the momentum for shifting quickly its workforce towards new innovations. CCS may react to recovery conditions with some lag, not only because of economic factors (lack of productivity gain, hence of profit) but also institutional factors (subsidies and policy incentives that make the CCS less cyclical than other industries).

As a consequence, CCS are more concerned about continuing education and learning schemes that may accelerate the implementation of innovation by granting its workforce with a blend of creative, digital, managerial and entrepreneurial competences. The ‘Halland Model’ as experienced in Sweden (Gustafsson 2011; Gustafsson and Rosvall 2008), whereby heritage conservation benefit from resources in other sectors (the adaptive reuse of heritage building providing the preservation of cultural values indirectly, not only as a cultural objective) is an indication of the need for innovative cross-sectoral schemes, cross-fertilisation between cultural activities and economic or social needs. Unemployed construction workers and apprentices were trained in traditional building techniques and gained proficiency under the supervision of skilled craftsmen and conservation officers.

A new wave of innovation is always correlated to adaptive programming of curriculum and training programs. As new needs emerge in the CCS, a new educational market offers teaching and learning opportunities for CCS’ stakeholders. Although the movement is a global one and indicates the common focus of the education sector to face those challenges, the CCS rely to a very large range of artistic and cultural actors. Multidisciplinary programs are needed, as well as international and intercultural curriculum that reflect the global scope of the new wave of innovation. Keeping an eye on what is happening internationally walks hand in hand with valorising local resources and re-interpreting them according to contemporary local needs. Therefore, there is more than ever a necessity to contribute to local flourishing and well-being while cherishing and reinforcing solidarity and connections with peers worldwide. It is hard to determine how the CCS will be structured in the long-run, and how the education system will adapt itself to that evolution (peer-to-peer, blended learning, school of arts, arts management program, educational niches, etc...).

## Cultural entrepreneurship: a peculiar branch of the classic entrepreneurship theory

While the profound disruption to cultural life and livelihoods caused by COVID-19 is palpable, the current crisis might be also perceived as an opportunity to trigger efforts to increase cultural resilience and sustainability, as well as harness the universality and power of creativity. As the preceding section has described, economic history is filled with many examples of crisis as triggers for innovation and creativity that contribute to cyclical waves of creative destruction. Such processes of technological creativity are shaped in the same way as artistic creativity.

The sector of cultural built heritage and conservation is no exception. The COVID-19 has impacted the sector in different ways (ICOMOS 2020). Heritage economics used to define heritage conservation as a two-fold market. The real estate market of historic buildings is supply-driven, since the uniqueness of the heritage hinders easy substitutes to the goods which are exchanged. Besides, the most cultural of heritage buildings are seldom exchanged on the market, and heritage monuments are totally excluded from the real estate market. The question is to which extent a COVID-19 disruption in the real estate market still impact activities in the sector of cultural built heritage. On the other hand, there is demand-driven market of the use of the heritage. Cultural tourism is one of the main segment of this market, and needless to say that cultural heritage is impacted by the collapse of travelling and visiting during the crisis. The question here relies on how cultural entrepreneurship could enhance creative and innovative ways of developing cultural tourism in post-crisis time?

According to Block et al. (2017), a debate on how the role and importance of entrepreneurship for society depends on the definition of entrepreneurship has begun, and has received increasing research attention. The definition which we are interested in exploring in this paper is the one provided by Schumpeter where entrepreneurship is the act of turning new ideas into marketable goods and services. Thus, as indicated before, the process of transforming scientific invention into market innovation.

‘By and large, researchers are now realising that the desired benefits from entrepreneurship are mostly generated by a small number of innovative, high-growth ventures, whereas the vast majority of new ventures only experiencing moderate growth in terms of employment and turnover, if they survive at all.’ (Block et al. 2017, 62). It is exactly here where the role of the cultural and creative sectors become crucial.

A systematic literature review conducted in 2016 by Andrea Hausmann and Anne Heinze and in 2020 by Nevena Dobreva and Stanislav Ivanov shows that cultural entrepreneurship became a research topic starting from 2001 and the number of scientific publications increased especially since 2006. The former scholars (Hausmann and Heinze 2016) analysed 50 papers published between 1996 and 2015. Their research highlighted that although cultural entrepreneurship is the most commonly used term in the reviewed literature, ‘a general theory defining the respective term is often missing’. The scholars argue also that ‘characteristics of entrepreneurship discussed in classic literature, such as entrepreneurial opportunity, innovation, and novel combinations, as well as the creation of an organisation, are partly included in an overall definition of arts, creative, and cultural entrepreneurship’. Furthermore, the scholars stress the fact that ‘in many cases, no distinction is made between entrepreneurs, managers, self-employed workers, freelancer, and owner-managers, as general entrepreneurship theory usually does’ (Hausmann and Heinze 2016). Based on their research, the scholars conclude that classic entrepreneurship narrative is rarely tangible in the CCS. While the latter scholars analyse 131 papers published between 1982 and 2019. Their qualitative thematic analysis of the publications enabled them to identify eight research domains under the emerging field of cultural entrepreneurship namely: ‘“Characteristics and motivation of entrepreneurs”, “Business models”, “Audience development”, “Use of information and communication technologies”, “Urban development”, “Public policy”, “Incubators and clusters” and “Entrepreneurial education”’ (Dobreva and Stanislav 2020).

Cultural Entrepreneurship is studied by management, business, cultural studies, cultural economy, sociology and anthropology scholars. Regardless of the discipline, entrepreneurship theory is the common denominator for the provided definitions. For instance, characteristics of general entrepreneurship theory such as exploration, assessment, and harnessing of an entrepreneurial opportunity; innovation both perceived as novel ideas, ways of doing, and the ability of bringing innovation into the market; and the creation of an organization. Under the same theoretical framework, scholars do also investigate the virtues of the cultural entrepreneur and the motivation behind launching his/her entrepreneurial journey. In a true Schumpeterian perspective, this includes the capacity to manage resources, the organisational power, the talent of persuasion, the strength of their collaborative ways of working; the visionary vision, risk-taking and adventures traits, knowledge and sensitiveness to the artistic process, capability of interpreting, transforming and transmitting new goods and products without undermining their cultural and creative intrinsic value.

The management discipline has been focused on project, risk, resources and management culture aspects. The business discipline looks more into value creation and delivery (enterprising) through which tools (innovative business models). Cultural studies emphasize the cultural and creative values while scholars in cultural economics focus on the embodied and yielded cultural and economic values. Finally, sociology exploits the Bourdieu framework of the forms of capital in order to understand how cultural entrepreneurship is characterized by a collaborative economy which mobilizes the social, cultural and symbolic capital (Scott 2012). Different titles are attributed to a person launching a new activity, product, service or organisation within the cultural and creative sector. While cultural entrepreneur is frequently found in literature nowadays, one can also find cultural capitalist, *culturepreneur*, arts entrepreneur and creative entrepreneur. A common denominator is the fact that individuals –sometimes as isolated and rejected innovators- provides a bridge between micro-ideas to macro relevance and impacts. In this meaning, cultural entrepreneurs contribute to the transition of the economy and society as a whole (Schumpeter 1968).

Nevertheless, not all scholars agree on depicting the cultural entrepreneurship as a voluntary choice but on the contrary, some refer to it as the activities carried out by self-employed freelancers and cultural and creative workers, who are forced by the precarious labour market conditions in the cultural sector to act as entrepreneurs (Ellmeier 2003).

To date, there is no agreed upon definition of cultural entrepreneurship. However, we would like to provide the following definition: Cultural entrepreneurship is a set of activities aimed at harnessing a cultural business opportunity. The novelty stands in being innovative in transforming cultural values into economic values. The process of creating new cultural expressions could be also interpreted as the business of transforming intangible values (performing arts, artistic creation, traditions and knowledge, etc. …) into tangible assets in the form of cultural capital. The process of creating new adaptive reuse of heritage buildings is about the business of transforming abandoned, underused or not in use cultural heritage into common goods which reflect needs and aspirations of the contemporary local community with respect to environment and social practices and interactions. By transforming the cultural asset, the cultural entrepreneur harnesses the existing cultural (tangible and intangible) and economic values and transform them into enhanced cultural, economic, social and environmental impacts, outcomes and benefits. For both processes, the cultural entrepreneur makes use of new skills and technologies to transform assets into innovative cultural services, goods, uses and organizational forms that generate financial revenues, positive societal impacts, and new creative and cultural markets.

Cultural entrepreneurship is gaining momentum nowadays because it elaborates on new organizational forms of business and finance of cultural activities. It revisits the role of the state (both central and local) and keeps into account the constraints that might impact on the performance and outcomes. It enables new ideas on the quantity and quality of resources—in particular financial resources in a framework where the role of public authorities may become limited and where forms of crowdfunding and other alternative ways of cultural finance are at stake. Cultural entrepreneurship also refers to the way cultural heritage initiatives are governed where the community—seen as the group of individuals that will be using heritage sites—is becoming leader, actor, and decision-maker.

Recently, just like other impact entrepreneurs, cultural entrepreneurs are also keen at ‘doing no harm’ to the environment as well. This is possible not only because of the visionary leadership and characteristic of the cultural entrepreneur but also thanks to the organisation behind him/her and to the adoption of strongly sustainable business models. Deploying these innovative tools is having a twofold impact: On one hand, it is attracting the attention of the public and private sectors alike. In a similar manner, it is stimulating public policies tabling and discussions around the role of cultural entrepreneurship in not only growth and job creation but also in humanizing our lived environment. However, not everyone is an entrepreneur and not all cultural entrepreneurs are equipped with the right toolkit. For this reason, cultural entrepreneurship is taking ground as an academic discipline aimed at studying, accompanying and empowering the cultural entrepreneur in his/her entrepreneurial journey. Nevertheless, scholars’ put emphasis also on the environment as an enabler/disabler of the cultural and creative activities.

## Would CCS’ professionals fancy an entrepreneurial journey? A survey

The magnified precariousness of the CCS coupled with the multiple repercussions (economic, professional, social, emotional, etc. …) on the people working in the CCS caused by the pandemic made us go back again to Schumpeter’s creative destruction theory and explore agile and resilient strategies based on existing resources. In order to make ends meet, we believe that there is a need to develop and experiment new sustainable and circular business models in tandem with addressing the CCS from a cultural entrepreneurship perspective. This recovery strategy is two-fold: First, delivering innovative business tools which are adapted to the ongoing trends of sustainability, circular economy, and CCS-oriented managerial techniques; Second, coaching and mentoring CCS’s decision-makers towards better entrepreneurship.

As far as the cultural built heritage is concerned, it entails the use of specific business models, such as the circular business model for the adaptive reuse of cultural heritage developed by the authors under the framework of H2020 project CLIC^2^ (Saleh et al. 2020; Ost and Saleh 2021). Such adapted business models are helpful to embed conservation projects or adaptive reuse projects within a broader documentation of the place, relying on elaborated participatory approach, consistent with UNESCO s Historic Urban Landscape recommendation. It also entails coaching and mentoring conservation specialists, architects, and art historians towards managerial attitudes (not just management tools) and creative behaviour.

However, before describing what an entrepreneurial journey entails, a mapping of the needs and challenges was deemed necessary. For this reason, a survey addressing professionals from the CCS interested in starting an entrepreneurial journey was launched.

Under the framework of the cultural entrepreneurship project C-SHIP at *ICHEC Brussels Management School*, the survey was open from 19 October until 4 November 2020. It was available on ICHEC’s Social Media (Facebook, Twitter and LinkedIn); circulated to the authors’ professional network via email; and kindly hosted by creatives unite, the artists and creatives community COVID-19 resource platform operated by the European Creative Hubs Network and the Goethe-Institut as part of Creative FLIP.^3^ 100 respondents participated in the survey from various age-rages.

### Composition of the sample

**Fig. 3:**
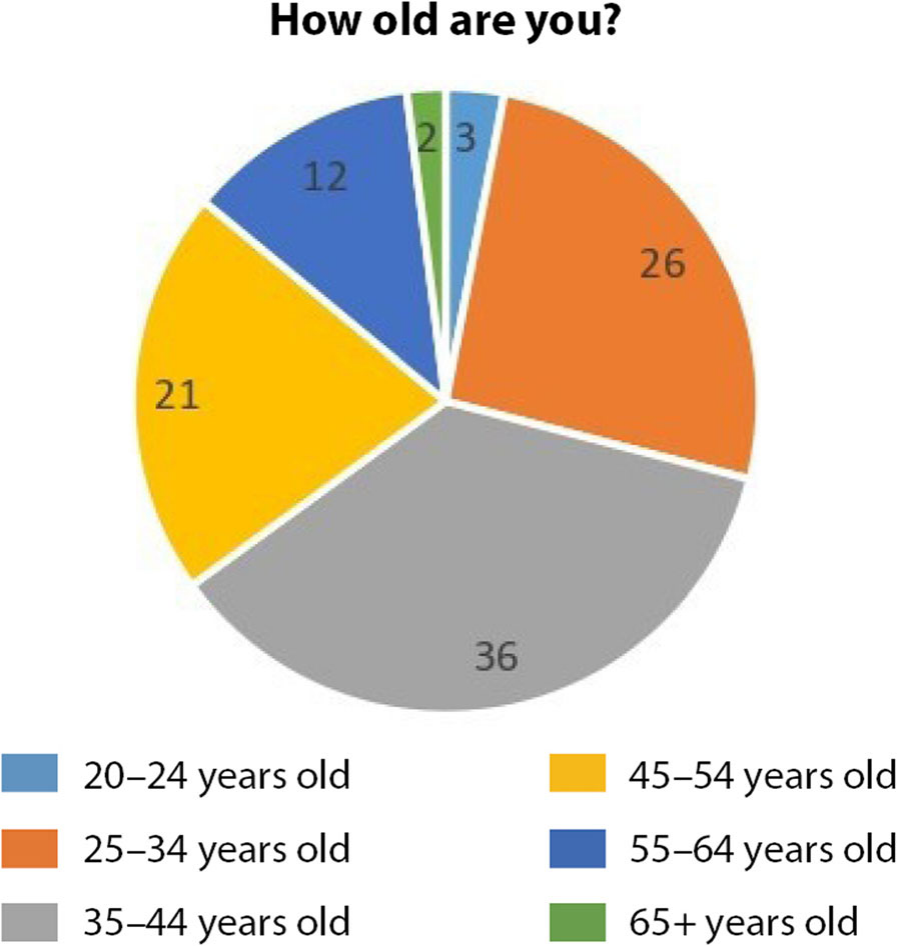
respondents age-range distribution (Source: the authors)

**Fig. 4:**
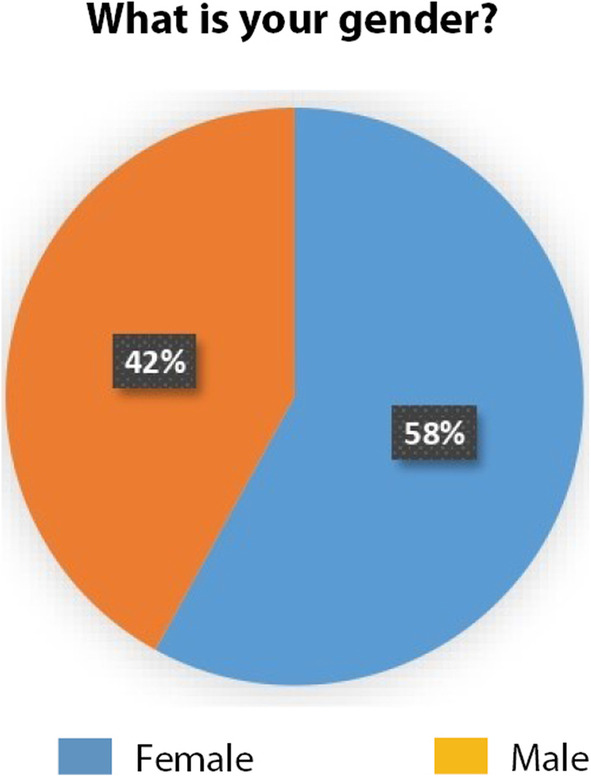
Respondents gender distribution (Source: the authors)

58% of respondents were females and 42% were males representing 27 countries (Australia, Austria, Belgium, Brazil, Czech Republic, Denmark, France, Germany, Greece, Ireland, Israel, Italy, Mexico, Malta, Morocco, the Netherlands, Norway, Palestine, Poland, Portugal, Romania, Spain, Sweden, Turkey, United Arab Emirates, USA, United Kingdom) (Figs. 3 and 4).

**Fig. 5:**
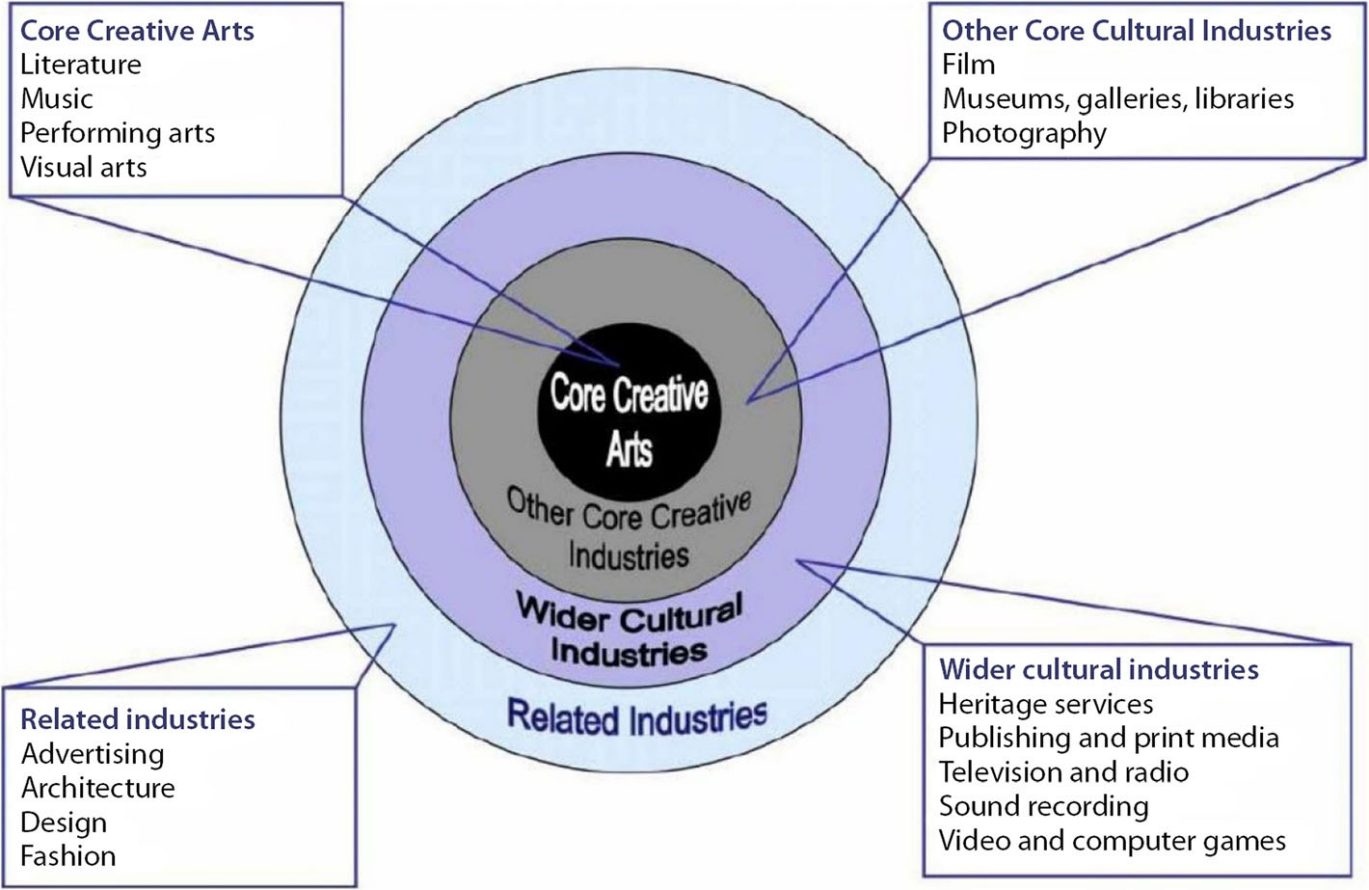
The concentric circles model of the cultural industries (Source: Throsby 2001)

**Fig. 6:**
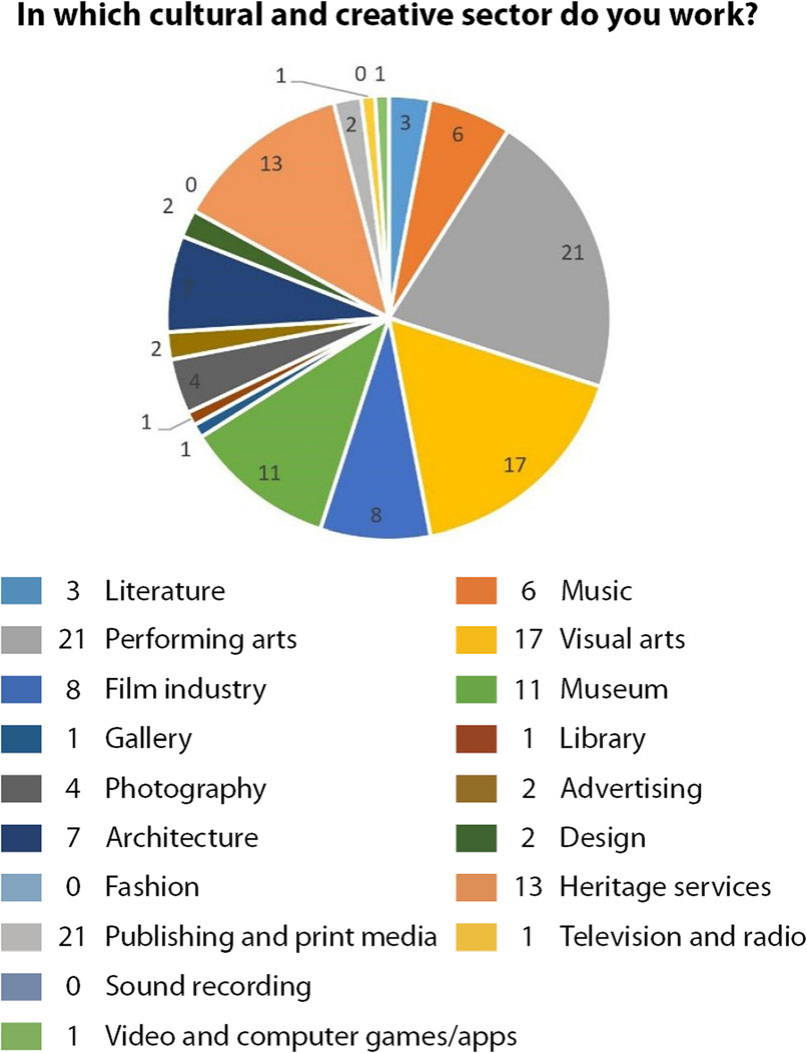
respondents’ professional sectors distribution (Source: the authors)

The respondents were asked to select the cultural and creative sector in which they work and were provided with a list based on the concentric circles model of the cultural industries (Throsby 2001) (Fig. 5) except for fashion and sound recording for which no answers were registered. From the core creative arts category, performing arts was the highest participating sector group with 21% followed by 17% of respondents from the visual arts sector. While only 6% of respondents were from the music sector; and 3% from the literature sector. From the category other core cultural industries, the museum sector was the highest participating sector group with 11%; followed by 8% of respondents from the film industry; 4% from the photography sector; and only 1% of respondents from both gallery and library sectors. As per the related industries, architecture was the highest participating sector group with 7% followed by 2% of respondents from both the advertising and design sectors. Finally, the wider cultural industries category registered heritage services as the highest participating sector group with 13% followed by 2% of respondents from publishing and print media sector and only 1% of respondents from both television and radio and video and computer games/apps sectors (Fig. 6).

**Fig. 7:**
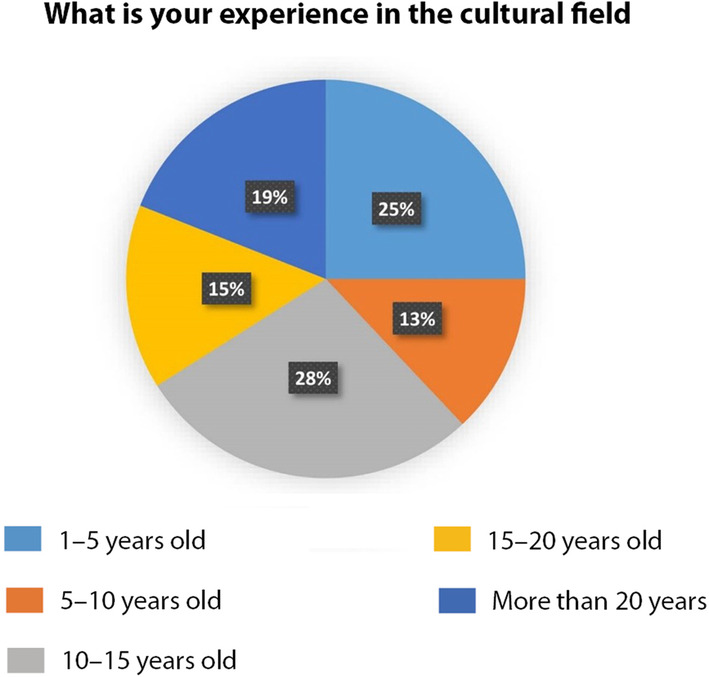
respondents’ experience (Source: the authors)

In terms of experience in the cultural field (Fig. 7), 28% of respondents represented experienced professionals of 10–15 years of experience, 19% represented seasoned professionals of more than 20 years of experience and 15% represented senior professionals of 15–20 years of experience, 13% represented mid-level professionals of 5–10 and 25% represented junior professional of 1–5 years.

**Fig. 8:**
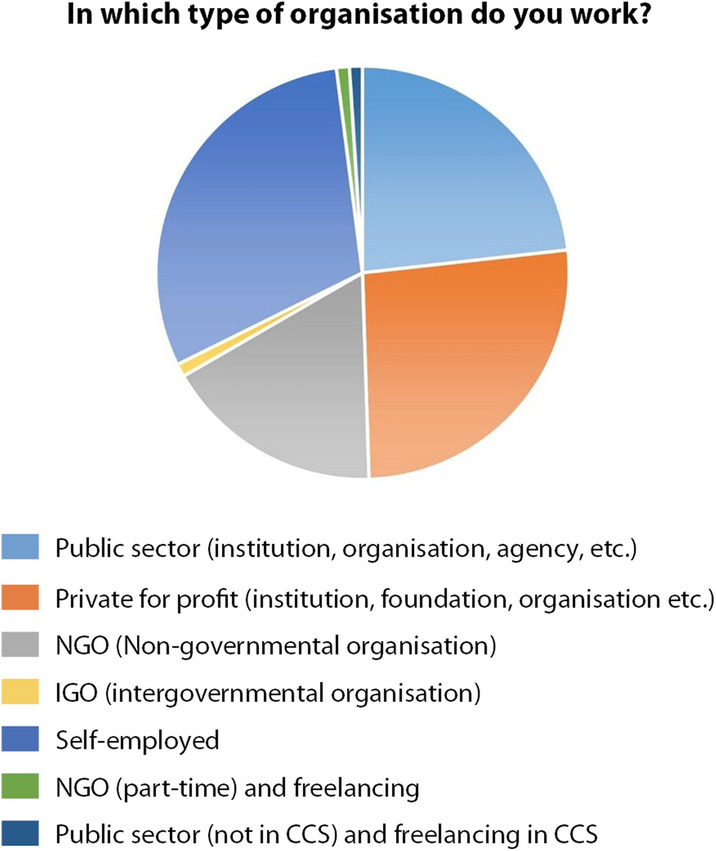
In which type of organisation do you work? (Source: the authors)

When asked about the organization in which they work (Fig. 8), 30% declared to be self-employed; 26% working for the private for profit, 23% employed in the public sector; 17% working in Non-Governmental Organizations and only 1% working in an Intergovernmental Organization. Since we intended to map the variety of existing organizations, we added also the option ‘other’. Two additional categories were added by two respondents identifying themselves as artists, as follows: ‘part-time in an NGO and freelancing in the CCS’; and ‘part-time in a public organization not from the CCS (school) and freelancing in the CCS’. Although these two contributions do not add up to the organization category, they do instead emphasis the precariousness of cultural and creative professionals. On this same note, one of the two artists commented: ‘Your questionnaire doesn’t accommodate multiple professions, and sources of income, which is key to our sector’.

**Fig. 9:**
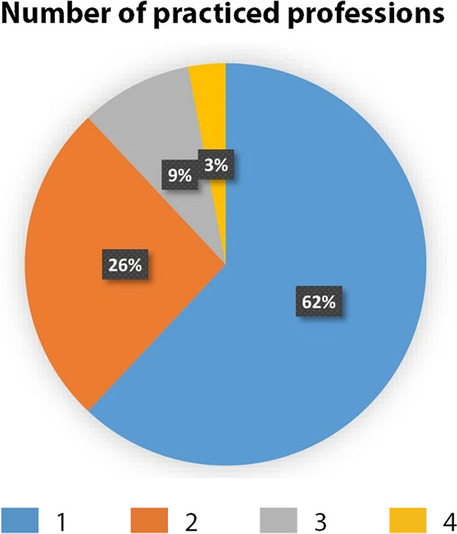
Number of practiced professions (Source: the authors)

When asked about their practiced profession (Fig. 9), 62% of respondents declared one profession, 26% practiced two, 9% practiced three and 3% practiced four different jobs. This shows the different hats a creative professional has to wear in order not to abandon the artistic and creative dream while making a living.

**Fig. 10:**
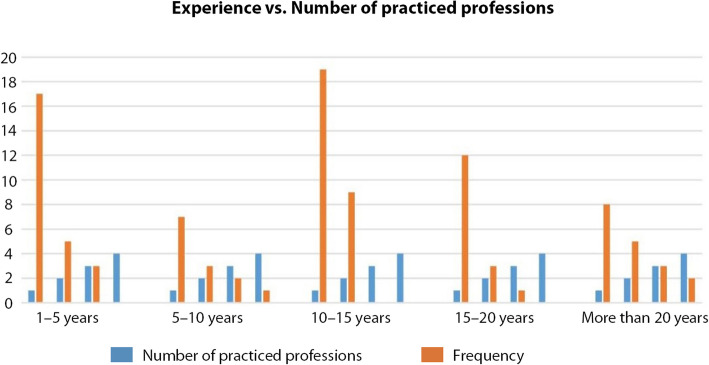
Experience vs. number of practiced professions (Source: the authors)

We tried to investigate whether there is a correlation between experience and the number of practiced jobs. Out of the 25 junior professionals (1–5 years), 17 practiced one job, 5 practiced two jobs, and 3 practiced three jobs (Fig. 10). Out of the 13 mid-level professionals (5–10 years), 7 practiced one job, 3 practiced two jobs, 2 practiced three jobs, and 1 practiced four jobs. Out of 28 experienced professionals (10–15 years of experience), 19 practiced one job and 9 practiced two jobs. Out of the 16 senior professionals (15–20 years), 12 practiced one job, 3 practiced two jobs and 1 practiced three jobs. Finally, out of the 18 seasoned professionals (more than 20 years), 8 practiced one job, 5 practiced two jobs, 3 practiced three jobs and 2 practiced four jobs. The survey demonstrates that no matter how experienced a CCS professional is, s/he still experience precarious jobs and work status. This is related to the already existing uncertainty of demand- and consequently to available jobs- and to the subjective self-evaluation for which ‘workers care about originality, technical professional skill, harmony, etc. of creative goods and are willing to settle for lower wages’ (Caves 2000, 2). This also explains why Throsby debates that remuneration is not an adequate criterion to define a cultural and creative professional ‘because of multiple job-holding amongst artists and because some professional artists may receive little or no remuneration over significant time periods in their working lives.’ (Throsby 2010, 2018).

**Fig. 11:**
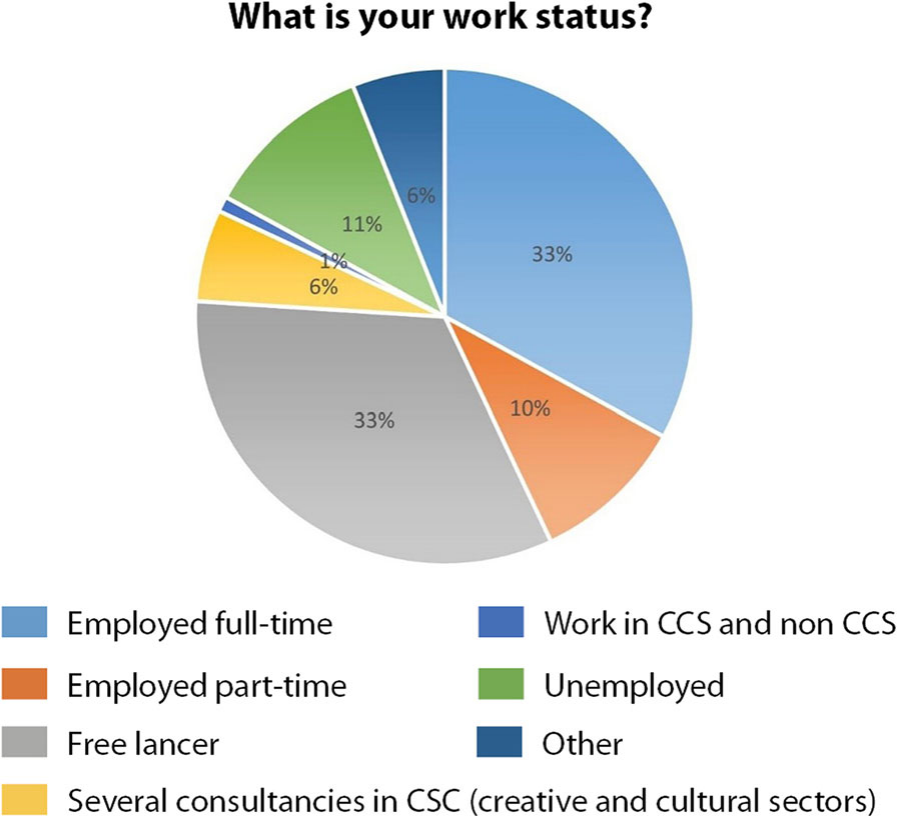
respondents’ work status (Source: the authors)

With reference to employment (Fig. 11), 30% is self-employed, 23% work in the public sector, 25% work in the private for profit sector, 14% work in the Not-for-profit and 1% in IGO while 7% answered other.

The majority of respondents were self-employed professionals who usually struggle by virtue of their precarious working conditions, which are currently magnified because of the pandemic. The answers are in line with the dataset of the Eurostat^4^ (Eurostat 2019) which shows that one third (33%) of the cultural workforce is self-employed across the EU Member States, compared with an average of 14% for the whole economy.

This means that, although these professionals find it important to upskill, they might not have the financial means to enrol in an upskilling course. This is why, reaching out to policy-makers and impact investors interested in supporting professionals from the CCS either by providing scholarships or investing in their course project is key in the process. Not only, funders have also to review their funding structure and priorities allowing for flexible and agile funding. Seen the current circumstances, it is not possible to play by the book and continue business as usual. Beneficiaries are always requested to adapt and be inventive, can’t funders follow the same path and revise their evaluation and impact criteria?

### Impact of COVID-19 on CCS’ activities

The aim of the survey was to assess the impact of the COVID-19 on CCS’s artistic business and activity in tandem with exploring interest in upskilling in sustainable and agile business management. As described here above, the cyclical framework of economic growth brings an endogenous incentive during the upward stage, and a constraint during the downward stage. All industries and economic activities are by definition cyclical, and dominantly pro-cyclical (the activity increase and decrease with the general economic conditions), otherwise, the cycle would not be like it is. Nevertheless, all economic activities are not timely correlated: some move earlier (leading), some are coincident to the main cycle, and some move later (lagging). As it has been explained previously, CCS are expected to be very sensitive to any change in the general economic conditions (GDP rate of growth), partly because they are low capital-intensive, their activity do not benefit from high productivity gains, and the uniqueness of their product and services (two performances of Beethoven’s symphony will be different). In other words, the CCS can be highly disrupted by a major upturn/downturn of economic fluctuations, and severely impacted in a crisis.

**Fig. 12:**
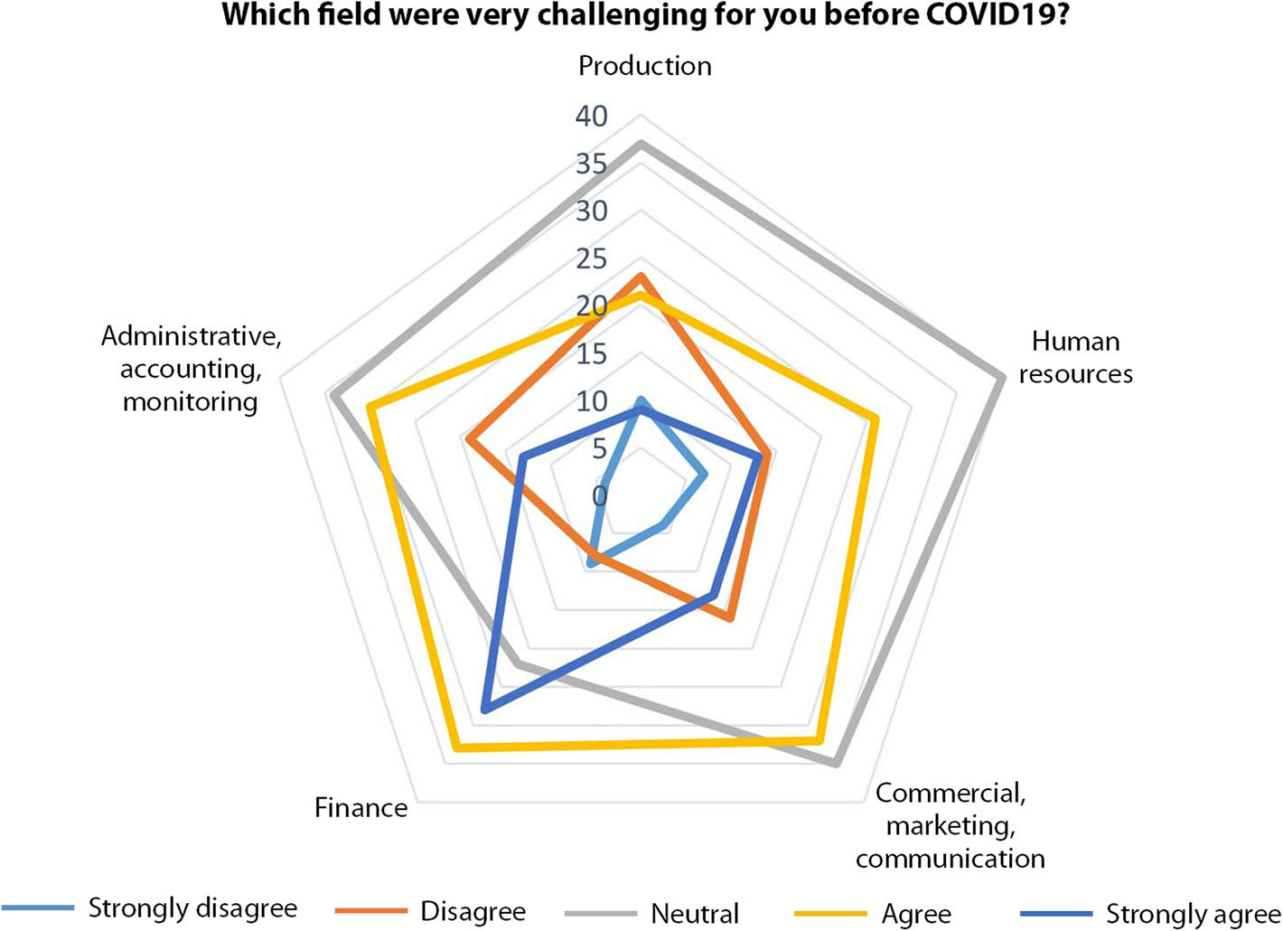
challenging fields before COVID-19 (Source: the authors)

The next questions of the survey aimed to assess such impacts at the light of the expected sensitivity. Figures 11 and 12 show different management fields challenges before COVID-19. Not surprisingly the financial and commercial sides ranked highly. Noteworthy to mention that operational tasks were also at stake before the crisis. Those indications are in line with the expressed needs of the CCS in terms of continuing education and support.

**Fig. 13:**
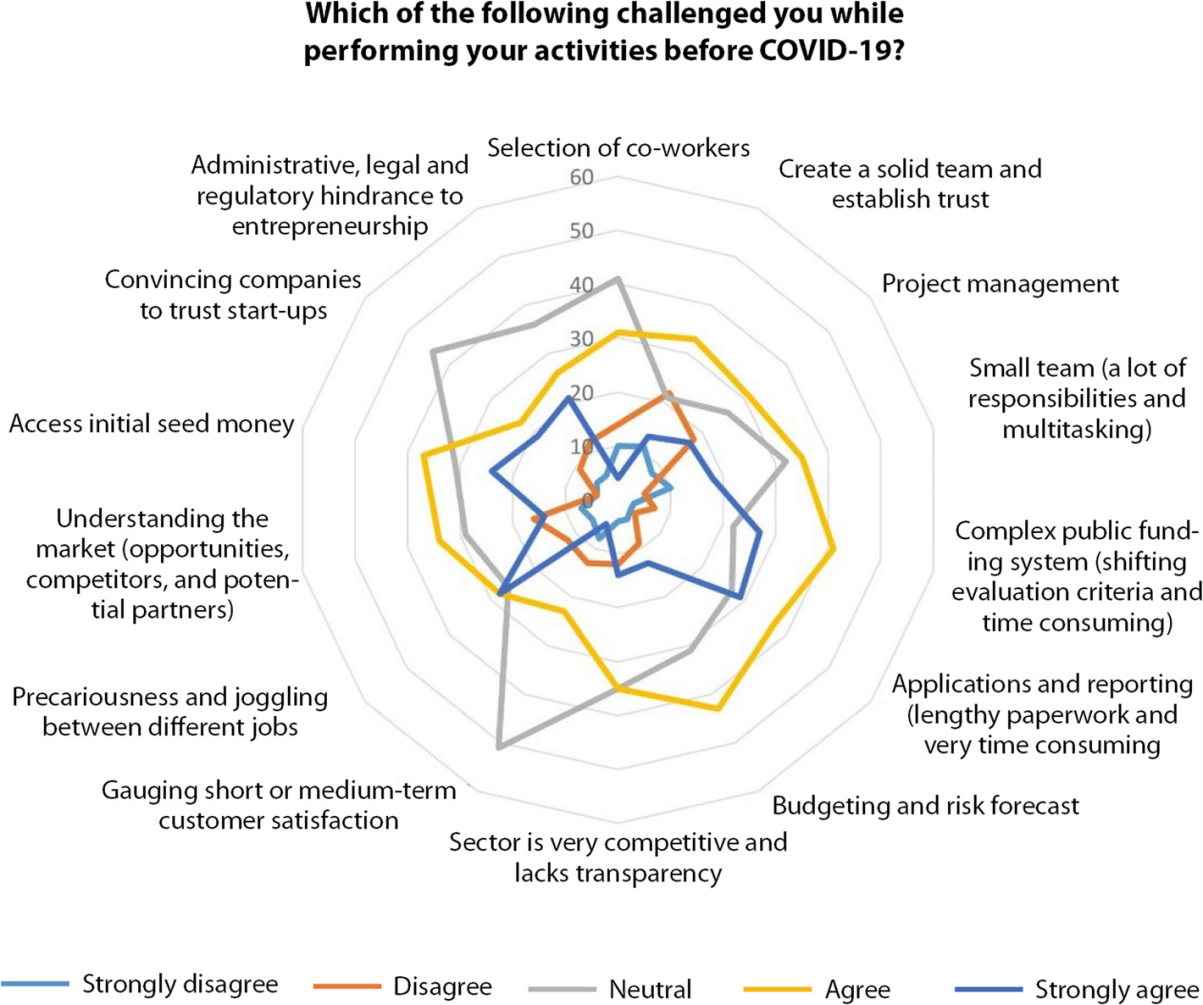
challenging aspects while performing activities before COVID-19 (Source: the authors)

Figure 13 depicts the ex-post assessment of COVID-19 impacts on the CCS activities. It shows how difficult the communication was during the crisis, with no surprise when we realize how erratic was the public communication at the same time, because of the so many unknowns and uncertainties about the new virus. The lagging communication in combination with pending responses from both funders towards the CCS and cultural institutions towards artists and freelancers crippled the collective reflection on alternative solutions. It is positive to underline that CCS’ professionals are eager to exploit new technologies for creativity and culture. It is clear that the global crisis is accelerating the mind shift and enriching it within the CCS with an artistic and creative interpretation of how to make the leap while keeping the artistic quality and measuring success and self-attainment differently. The pandemic speeded also a power shift within the sector between established cultural institutions and freelances. During the pandemic, we witnessed a growing independence of tech savvy artists, artistic collectives/companies and producers envisioning their work on digital platforms instead of physical ones coupled with autonomy in managing one’s own public. As the recent report of ITEM puts it forward, the autonomy path which started before the pandemic and was enhanced by it is twofold; physical and financial (Polivtseva 2020).

**Fig. 14:**
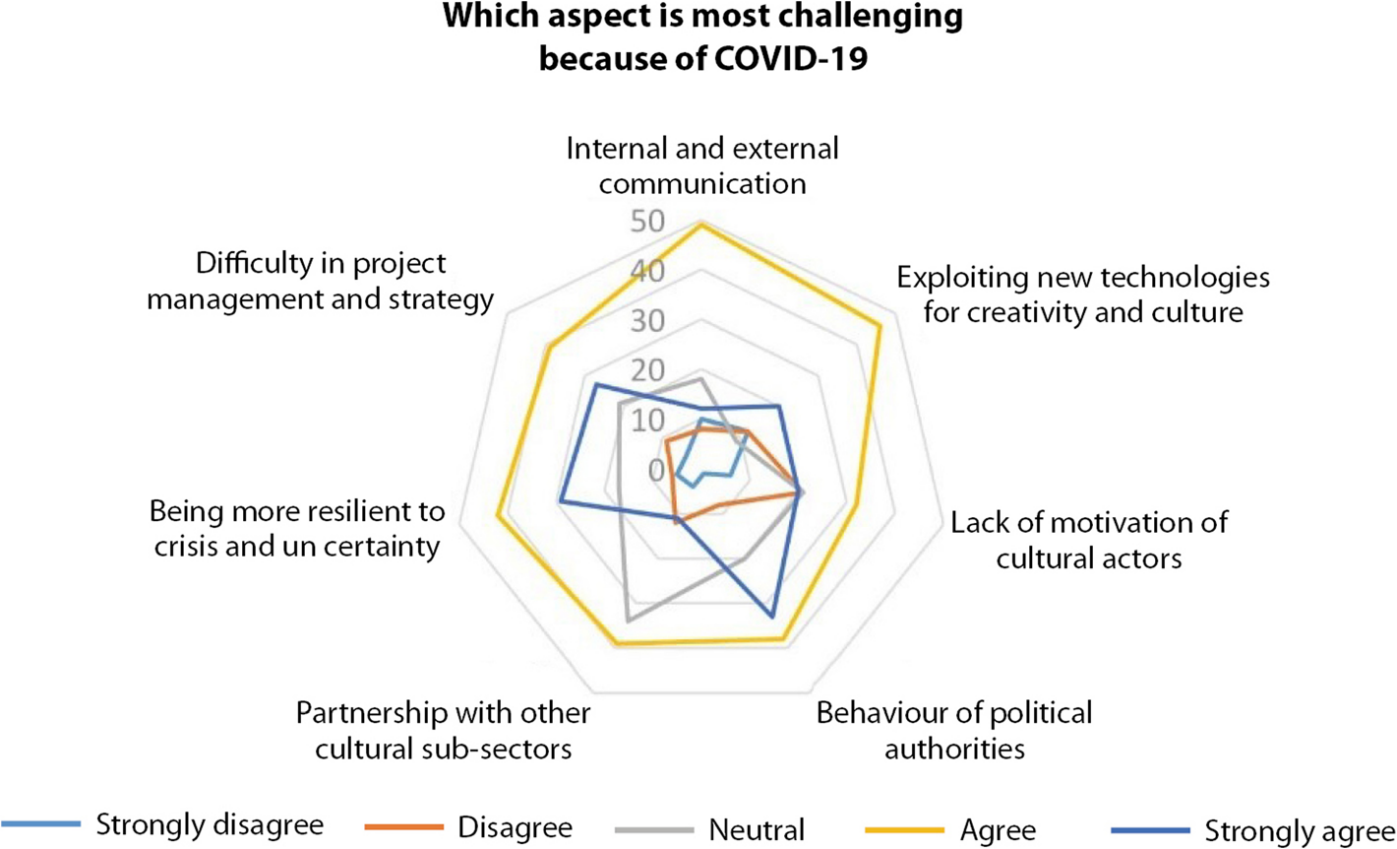
challenging aspects because of COVID-19 (Source: the authors)

Figure 14 gives some indication about the needs a training programme would address. Focus is on the learning of what makes the world change, on which uncertainties we live with, and on how we can improve our behaviour to create and innovate. Clearly, the sample stresses on the big issues that the long wave explanation of the current crisis reveal: sustainability, innovations, peer-to-peer exchanges. Day-to-day operational tasks are less mentioned, not because the current needs are fulfilled already, but because the learning prioritizes heavy trends and not short-term techniques. Resilience is a very critical issue, and it is to be read above all from a work status angle since one third of respondents is self-employed. The recent report of the European Expert Network on Culture and Audio-visual which investigated the status and the working conditions of artists and creative professionals showed that a consistent number of freelancers from the sector haven’t been able to take advantage of any of the ad-hoc public rescue packages for various technical/bureaucratic reasons (EENCA 2020, 51). Moreover, the figure shows that there is a growing difficulty of project management and strategy. This is without doubt due to the uncertainties but also to new incumbent issues related to digital environment and copyrights. While fees were fixed based on live performances and an estimated audience in venues (theatres, museums, cultural centres, etc. …), broadcasted performances, artistic digital innovations and interactions reach out to a wider public. This poses a challenge on what would be the correct remuneration for all the professionals involved in the creative piece of work (artists, curators, authors, technicians, etc. …).

**Fig. 15:**
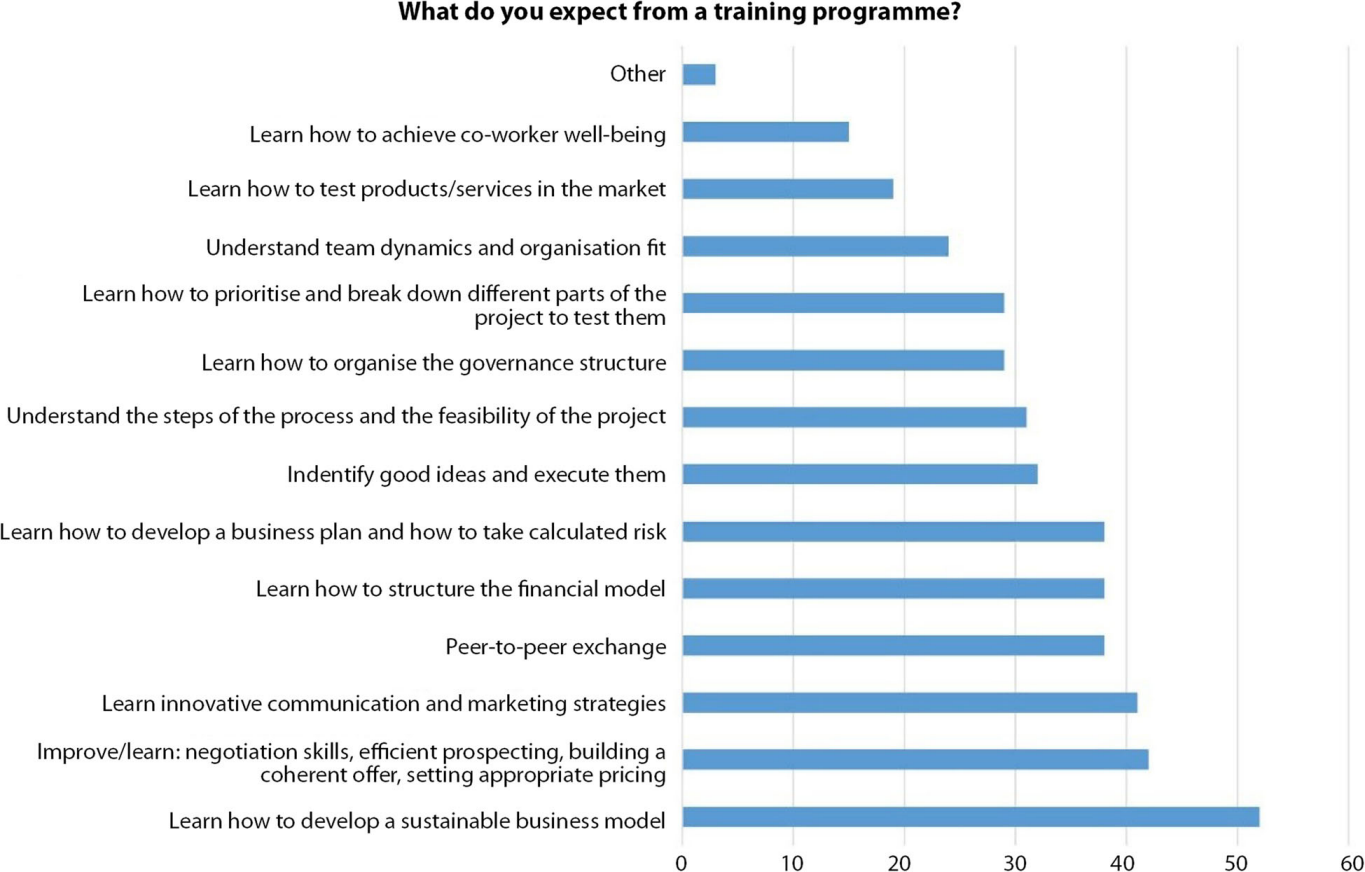
What do you expect from a training programme? (Source: the authors)

Finally, respondents are mostly interested in learning how to develop sustainable business models because by its intrinsic nature, the sector is content-driven and often lacks business management skills (Fig. 15). Such skills are crucial nowadays to access funding mechanisms (Kern 2020).

## Conclusions

The COVID-19 sanitary crisis has been a global challenge for the social and economic life, and a strong impetus in the long wave-related transition period that the global economy experiences since the end of last century. Never in the past have new technologies been so quickly available on a global scale, offering opportunities for all sectors of activities, including the cultural built heritage. Most of these technological and organizational innovations appear in the cultural and creative industries, taken as a broad and generic field of human activities. The development of new COVID-19 vaccine is an example of how fast and efficient the international scientific research and collaboration could perform today. But by the same token, the current long wave-related transition period is also a time of uncertainty, destruction of past production and consumption patterns, global recession, and major political and social disruptions. The paper aimed to emphasize that the current transition period is timely related to the questioning of modern heritage conservation. Are heritage values, and our current awareness towards the tangible and intangible heritage enough today to preserve our world heritage in troubled times? Is there no need for a change in paradigm, in the perspective of new ways of living, and sustainable conservation in particular in urban settings? Such challenges were typical in upturns/downturns of long waves in history. The current COVID-19 sanitary crisis takes the role of catalyst in these changes today.

Back in 2006, KEA European Affairs was commissioned by the EU to measure and capture direct and indirect socio-economic impacts of the Cultural and Creative Sectors (CCS) within the European Union. Its published report ‘the Economy of Culture’, is the first comprehensive study attempting to map the CCS contribution in connection to growth, competitiveness, jobs creation, sustainable development, and innovation at the EU level (KEA European Affairs 2006). Building on this first assessment, the European Commission adopted its first European Agenda for Culture in 2007 (European Commission 2007) and Eurostat published its first dataset of Cultural Statistics (Eurostat 2007). Since then, the CCS are perceived as key drivers of growth and job creation in the EU (European Commission 2013). The year 2021 is the International Year of Creative Economy for Sustainable Development. However, CCS are still far away from being sustainable. Based on its recent survey and in depth interviews, the *International network for contemporary performing arts* provides a sharp framing of the major challenges to the performing arts sector which has a lot in common with the other CCS ‘to maintain the vibrancy and diversity of the performing arts sector and provide millions of highly skilled professionals with decent living and working conditions, policy-makers must take concrete steps to constructively address the multiplicity of issues at stake: social security, social benefits, unemployment status, remuneration, copyrights, funding structures, and many more’. (Polivtseva 2020, 7).

The challenge is enormous, as CCS stand at the interface between two realities. On the one hand, the world of culture with its diversity, uniqueness, pure creativity, universal values, qualitative and subjective dimension; while on the other hand, the world of economic transition with its new technologies, new needs and behaviours, new allocation of resources, new values, new quantitative models and innovative tools. Both realities face each other in the new framework of sustainable development, questioning the past and building the future. Despite the size of the challenge, still the current situation is not a unique one. It is something in fact recurrent in the history of development, unfolding in spurts, in similar not identical stages, in lasting and recurrent innovations. Such transition must be accompanied, not only by public policies, but by the emergence of new educational markets and tools which will allow cross-fertilization between cultural, social and economic projects and actors.

Our modest survey demonstrates that the respondents are interested in embracing a journey of social and cultural entrepreneurship with agile ways of working and systemic and inclusive approach to the creative economies. While the creativity remains high in the sector, there is a growing need to enable cultural professionals to fulfil financial sustainability and resilience. Three interrelated management upskilling pillars are crucial at this stage: agile and exponential organization, human and cooperative organization, and sustainable and positive impact organization.

The magnified precariousness caused by the pandemic coupled with multiple employment status and low remuneration in relation to the dedicated time and in comparison, to other sectors, calls for urgent measures.

Policymakers need to transform the sporadic and fragmented good practices at the EU level into a long-terms vision aimed at improving the work conditions of CCS professionals; facilitate their access to funding; and invest in research and professional training.

## Data Availability

All data generated or analyzed during this study are included in this published article.

## Abbreviations

CCS: Cultural and creative sectors
EU: European Union
IGO: Intergovernmental organizations

## Footnotes

1 This paper builds on the concentric circles model of the cultural industries (Throsby 2001).

2 https://www.clicproject.eu/

3 https://creativesunite.eu/

4 Eurostat (2019), culture statistics, P.70. European Union. Available at: https://ec.europa.eu/eurostat/documents/3217494/10177894/KS-01-19-712-EN-N.pdf/915f828b-daae-1cca-ba54-a87e90d6b68b
